# Editorial: Women in science: Genetics

**DOI:** 10.3389/fgene.2022.1038317

**Published:** 2022-10-03

**Authors:** Jaira Ferreira de Vasconcellos, Malak Abedalthagafi, Silvia Calo, Rana Dajani, Zodwa Dlamini, Bertha Hidalgo, Carine Le Goff, Aparna Vasanthakumar

**Affiliations:** ^1^ Department of Biology, James Madison University, Harrisonburg, VA, United States; ^2^ Department of Pathology and Laboratory Medicine, Emory University, Atlanta, GA, United States; ^3^ Pontificia Universidad Católica Madre y Maestra, Santiago de los Caballeros, Dominican Republic; ^4^ Department of Biology and Biotechnology, Faculty of Science, The Hashemite University, Zarqa, Jordan; ^5^ SAMRC Precision Oncology Research Unit (PORU), DSI/NRF SARChI Chair in Precision Oncology and Cancer Prevention (POCP), Pan African Cancer Research Institute (PACRI), University of Pretoria, Hatfield, South Africa; ^6^ Department of Epidemiology, University of Alabama at Birmingham, Birmingham, AL, United States; ^7^ Université Paris Cité and Université Sorbonne Paris Nord, INSERM U1148, Laboratory of Vascular Translational Science, Bichat Hospital, Paris, France; ^8^ AbbVie, North Chicago, IL, United States

**Keywords:** genetics, genomics, genome-wide association studies, genetic diversity, cancer, development

The reality of the workforce in Science, Technology, Engineering, and Mathematics (STEM) fields is still that women remain significantly underrepresented. Women obtain more than half of the undergraduate degrees in biology, chemistry, and mathematics in the United States and constitute about half of the American workforce. However, the American Community Survey from the United States Census Bureau has shown that women only held approximately 30% of STEM jobs in 2019 (Census, 2019). Of interest, the percentage of women pursuing STEM education is higher in the Middle East in comparison to the West (Study International, 2019), and according to the Organization for Economic Cooperation and Development women test better and feel more comfortable in mathematics than men in Jordan, Qatar, and the United Arab Emirates (Khazan, 2014). However, worldwide women continue to hold more junior positions in science, earn significantly less and do more unpaid work than their male colleagues, and are overall less likely to be supported during their higher education training (Author Anonymous, 2011; Khazan, 2014; Sommerfeld et al., 2017). Some overarching factors to help explain the larger gender gaps include masculine cultures that leave women with a low sense of belonging, the lack of sufficient early experiences in these fields, and gender gaps in self-efficacy (Cheryan et al., 2017). Moreover, more than 50% of women reported personal experiences with gender-related bias in a 2010 survey from AAAS/L'Oreal, compared with 2% of men who responded to the same survey (AAAS, 2010). And more recently, Chatterjee and Werner reported that original research articles written by women as primary authors had fewer citations than original research articles written by men as primary authors and senior authors, especially when both primary and senior authors were women (Chatterjee and Werner, 2021), demonstrating the existence of gender-based differences in article citations that can directly impact professional trajectory and success.

To promote interdisciplinarity and worldwide representation from women in genetics research, Frontiers in Genetics launched the Research Topic *Women in Science: Genetics*. This Research Topic accepted 17 manuscripts including 15 original articles, 1 review, and 1 case report from a wide variety of research focuses and to this date has over 29,200 total views, and 5,600 article downloads. The breadth of content published includes cutting-edge research questions and techniques in genetics applied to a wide variety of research focuses. Among them, 4 great contributions were made to cancer research. While significant progress has been made in the research and development of novel therapeutic strategies and precision medicine approaches, a lot of variability in the response to treatments as well as overall survival and disease-free survival still exists. In addition, relapses and secondary malignancy are still largely resistant to the current treatment strategies and a better understanding of the tumors underlying molecular mechanisms is still needed. In this Research Topic, we show that Zhang et al. identified five crucial genes associated with breast cancer progression in both primary and metastatic cancer tissues, which may be novel potential targets for the treatment of breast cancer. Yu et al. stratified cervical cancer in two subtypes based on their 5-Methylcytidine RNA modifications and identified a novel gene expression signature that may be used for clinical risk assessment and/or targeted therapeutical strategies for cervical cancer. Li et al. developed a novel scoring model focusing on the interaction of immune infiltration and ferroptosis to predict the overall survival of ovarian cancer patients. Finally, Sun et al. focused on endometrial cancer and correlated a messenger RNA alternative splicing gene signature to the patient’s prognosis and the immune-tumor microenvironment.

Additional genetics analyses were the focus of 6 contributions that used, for example, datasets from genome-wide association studies or bulk and single-cell RNA-seq to investigate different aspects of women’s health, minority populations’ health, or human development. He et al. used summary datasets from genome-wide association studies in a Mendelian randomization analysis to estimate the causal relationship between birth weight and female-related phenotypes and diseases. They demonstrated that birth weight may play a role in women’s body mass index, menarche, decreased levels of adult sex hormone-binding globulin, and increased levels of bioavailable testosterone. Liu et al. used bulk RNA-seq ovary datasets and single-cell RNA-seq follicles datasets to determine molecular mechanisms underlying the ovarian reserve, and identified a gene expression signature that was highly correlated to the ovarian reserve of the primordial follicle pool. This gene signature has the potential to be used as clinical biomarkers for the prediction of women’s ovarian reserve and in the development of future fertility targeted interventions. Yin et al. focused on competitive endogenous RNAs, including long non-coding RNAs, circular RNAs, and messenger RNAs, and their potential role in the pathogenesis of endometriosis. High-throughput sequencing demonstrated hundreds of long non-coding RNAs and circular RNAs differentially expressed as well as over one thousand messenger RNAs differentially expressed in the ectopic endometria group compared to normal and eutopic endometria groups. These results elucidate novel aspects of the underlying molecular mechanisms of endometriosis. Xie et al. investigated genetic polymorphisms associated with vitamin D anabolism and women’s susceptibility for uterine leiomyomas, a common type of benign gynecological tumor. Remarkably, they found that the DHCR7 rs1044482 C > T polymorphism, a vitamin D anabolism-related gene, was associated as a risk factor for uterine leiomyomas. Moreover, Armstrong et al. performed a meta-analysis to investigate genetic contributors to stroke among African Americans with hypertension and identified 10 statistically significant and 90 suggestive variants associated with a stroke incident in this population. These are critical results that shed light on potential genetic determinants for stroke on hypertensive African Americans. And finally, Lin et al. investigated sex-specific differences in microRNA expression during human fetal lung development by microRNA sequencing and identified over 120 microRNAs that were expressed with a specific male or female pattern during human lung development.

The investigations of novel underlying molecular mechanisms continued to identify novel mutations, map different stages of development as well as investigate the regulatory system of a clinically-relevant microorganism. In a case report contribution, Zhang et al. identified novel mutations in antithrombin and coagulation factor II and their downstream clinically relevant reduced activity (protein deficiency) phenotypes. *In silico* analysis also demonstrated that these mutations may destroy the function and structure of the antithrombin and coagulation factor II proteins. With a focus on endometrium development, Zhang et al. used single-cell RNA sequencing to map the mice uteri from the regenerative endometrium stage to the maturational endometrium stage and investigate novel fundamental molecular mechanisms occurring in the transitional states of the endometrium during the estrus cycle. They demonstrated novel transcription factors associated with the differentiation path and that different stages of the estrus have a distinct cell composition. Furthermore, Cholo et al. investigated the role of the *Mycobacterium tuberculosis* KdpDE regulatory system alone and in association with the Trk K^+^-uptake systems transporters in modulating *in vitro* bacterial growth.

The genetic diversity and population structure of plant populations were investigated by two contributions that used microsatellite markers or genotyping by sequencing to identify single nucleotide polymorphisms, respectively. Brooks et al. investigated wild Aspalathus linearis, also known as rooibos, regarding their genetic diversity and population structure and demonstrated that wild Cederberg populations from the Western Cape are genetically distinct from the wild Northern Cape populations, which shows the critical need for appropriate conservation strategies that protect wild ecotypes. Omire et al. focus was on the investigation of genetic diversity and population structure of *Hyphaene compressa* or doum palm, a plant that grows in the Arid and Semi-Arid lands of Kenya. They demonstrated moderate genetic differences among the populations which may inform future breeding and conservation efforts. In addition, phenological and morphological traits to help support novel target genes for breeding efforts in plants were the focus of 1 contribution. Katoch et al. investigated Macrotyloma uniflorum (Lam.) Verdc, commonly known as horse Gram, for their quantitative trait loci associated with phenological and morphological traits. Their findings demonstrated four and seven quantitative trait loci for phenological traits and for morphological traits across different environments, respectively, a knowledge that may inform future breeding strategies.

Finally, Elbardisy and Abedalthagafi wrote a review article focusing on the history and challenges of women in genetics, where they highlighted the obstacles and contributions made by women in science with a particular focus on non-western women’s contribution to the field of genetics.

We sincerely thank all our contributors for sharing their work on the Research Topic *Women in Science: Genetics* and all the reviewers and editorial staff involved in this endeavor for their time, expertise, and insights.
